# De Novo Transcriptome Analysis of Plant Pathogenic Fungus *Myrothecium roridum* and Identification of Genes Associated with Trichothecene Mycotoxin Biosynthesis

**DOI:** 10.3390/ijms18030497

**Published:** 2017-02-25

**Authors:** Wei Ye, Taomei Liu, Muzi Zhu, Weimin Zhang, Haohua Li, Zilei Huang, Saini Li

**Affiliations:** State Key Laboratory of Applied Microbiology Southern China, Guangdong Provincial Key Laboratory of Microbial Culture Collection and Application, Guangdong Open Laboratory of Applied Microbiology, Guangdong Institute of Microbiology, No. 100 Xianlie Middle Road, Yuexiu District, Guangzhou 510070, China; yewei@gdim.cn (W.Y.); ltm840801@163.com (T.L.); zhumuzi@foxmail.com (M.Z.); Lihh@gdim.cn (H.L.); yaoyz@gdim.cn (Z.H.); lisn@gdim.cn (S.L.)

**Keywords:** *Myrothecium roridum*, plant pathogenic fungus, transcriptome, trichothecene biosynthesis, MAPK signal pathway

## Abstract

*Myrothecium roridum* is a plant pathogenic fungus that infects different crops and decreases the yield of economical crops, including soybean, cotton, corn, pepper, and tomato. Until now, the pathogenic mechanism of *M. roridum* has remained unclear. Different types of trichothecene mycotoxins were isolated from *M. roridum*, and trichothecene was considered as a plant pathogenic factor of *M. roridum*. In this study, the transcriptome of *M. roridum* in different incubation durations was sequenced using an Illumina Hiseq 2000. A total of 35,485 transcripts and 25,996 unigenes for *M. roridum* were obtained from 8.0 Gb clean reads. The protein–protein network of the *M. roridum* transcriptome indicated that the mitogen-activated protein kinases signal pathway also played an important role in the pathogenicity of *M. roridum.* The genes related to trichothecene biosynthesis were annotated. The expression levels of these genes were also predicted and validated through quantitative real-time polymerase chain reaction. *Tri5* gene encoding trichodiene synthase was cloned and expressed, and the purified trichodiene synthase was able to catalyze farnesyl pyrophosphate into different kinds of sesquiterpenoids.*Tri4* and *Tri11* genes were expressed in *Escherichia coli*, and their corresponding enzymatic properties were characterized. The phylogenetic tree of trichodiene synthase showed a great discrepancy between the trichodiene synthase from *M. roridum* and other species. Our study on the genes related to trichothecene biosynthesis establishes a foundation for the *M. roridum* hazard prevention, thus improving the yields of economical crops.

## 1. Introduction

*Myrothecium roridum* is a plant pathogenic fungus widespread in the soils of tropical and subtropical regions. *M. roridum* infects different economical crops, including soybean, eggplant, pepper, tomato, and cotton, decreasing their yields [1]. This fungus shows strong infectivity, and the spread of *M. roridum* infection is very fast. Currently, no effective treatment and hazard prevention measure for *M. roridum* exists. Trichothecenes are sesquiterpene compounds isolated from different kinds of fungi, including *Stachybotrys atra* [2], *Trichoderma brevicompactum* [3], and *M. roridum* [4]. Trichothecenes are the main plant pathogenic factors of *Fusarium graminearum*; the treatment of trichothecene mycotoxin with wheat could cause gibberellic disease, and a substantial amount of trichothecene mycotoxin was found in infected wheat ears [5,6,7,8]. However, the main pathogenic mechanism of *M. roridum* remains unclear. Elucidating the molecular mechanism for the pathogenicity of *M. roridum* by transcriptome sequencing is necessary to facilitate the prevention of diseases caused by *M. roridum*.

The genes related to the trichothecene biosynthesis in *Fusarium* species were identified [9], and the trichodiene synthase encoded by the *Tri5* gene was considered a key enzyme in trichothecene biosynthesis. The structural and mechanism analysis of engineered trichodiene synthase from *Trichoderma harzianum* has been investigated, demonstrating that this enzyme could catalyze the conversion of farnesyl pyrophosphate (FPP) to trichodiene [10]. The *Tri5*-deleted *F. graminearum* almost lost its infectivity toward wheat [7]. The *Tri6* gene, encoding transcriptional proteins, could regulate trichothecene mycotoxin biosynthesis [6]. The *Tri4* gene encoded trichodiene oxygenase, the *Tri11* gene encoded isotrichodermin C-15 hydroxylase. The *Tri12* gene encoded a kind of membrane transport protein responsible for the exportation of trichothecene mycotoxins [11]. The *Tri5* gene from *M. roridum* was inserted into pMD18T vector and sequenced [12], but this gene has not been heterologously expressed and enzymatic characterized. However, genes related to trichothecene mycotoxin biosynthesis in *M. roridum* have not been fully identified.

Transcriptome sequencing in different kinds of infected plants or pathogenic fungi was performed to investigate the pathogenic mechanisms of fungi [13]. In this study, the transcriptome of *M. roridum* was sequenced using the Illumina sequencing platform 2000. Genes related to trichothecene mycotoxin biosynthesis in *M. roridum* including *Tri4*, *Tir5* and *Tri11* were identified. This is the first attempt to characterize the complete transcriptome of *M. roridum* and reveal genes related to trichothecene mycotoxin biosynthesis, which would provide a useful theoretical guide for the hazard prevention of *M. roridum* toward economical crops. Moreover, the elucidation of genes related to trichothecene mycotoxin biosynthesis establishes a foundation for the prevention of crop diseases caused by *M. roridum.*

## 2. Results

### 2.1. Sequencing and De Novo Assembly

**Figure 1:**
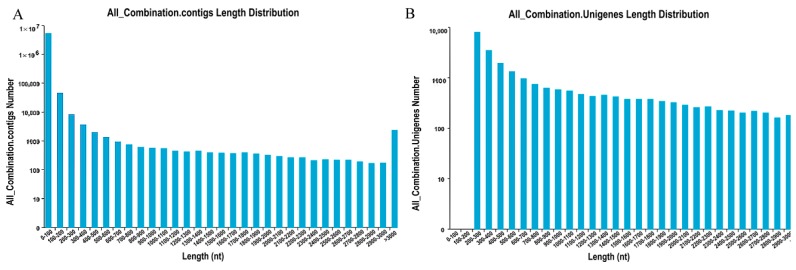
Raw data of *M. roridum* transcriptome: (**A**) the distribution of contigs in *M. roridum* transcriptome; and (**B**) the distribution of unigenes in *M. roridum* transcriptome.

In summary, 44,992,952 sequence reads with Q20 of 92.49% and GC percent of 57.57% were generated, and a total of 8,997,597,886 (8.79 GB) nucleotides were obtained (Table 1). Moreover, raw data were deposited in NCBI with accession number SRP080968. The Trinity software (http://trinityrnaseq.sourceforge.net/) was used to assemble short reads through a step-wise strategy, which yielded 5,247,474 contigs with a mean length of 39.83 bp (Figure 1A) and 25,996 unigenes with a mean length of 1094 bp (Figure 1B). A total of 2299 unigenes with lengths greater than 3000 bp were found, indicating the abundance of secondary metabolites in *M. roridum.*

### 2.2. Functional Annotation and Classification

All the unigenes were annotated by public databases, including Nr, Nt, Swissprot, KEGG (Kyoto Encyclopedia of Genes and Genomes), COG (Cluster of Orthologous Groups of proteins), GO (Gene Ontology Consortium); 18,199 unigenes were annotated, while 7797 unigenes remained unannotated. A total of 18,160 unigenes matched sequences in the Nr databases. Furthermore, the unigene number of *M. roridum* annotated in different public databases is shown in Table 2.

**Figure 2:**
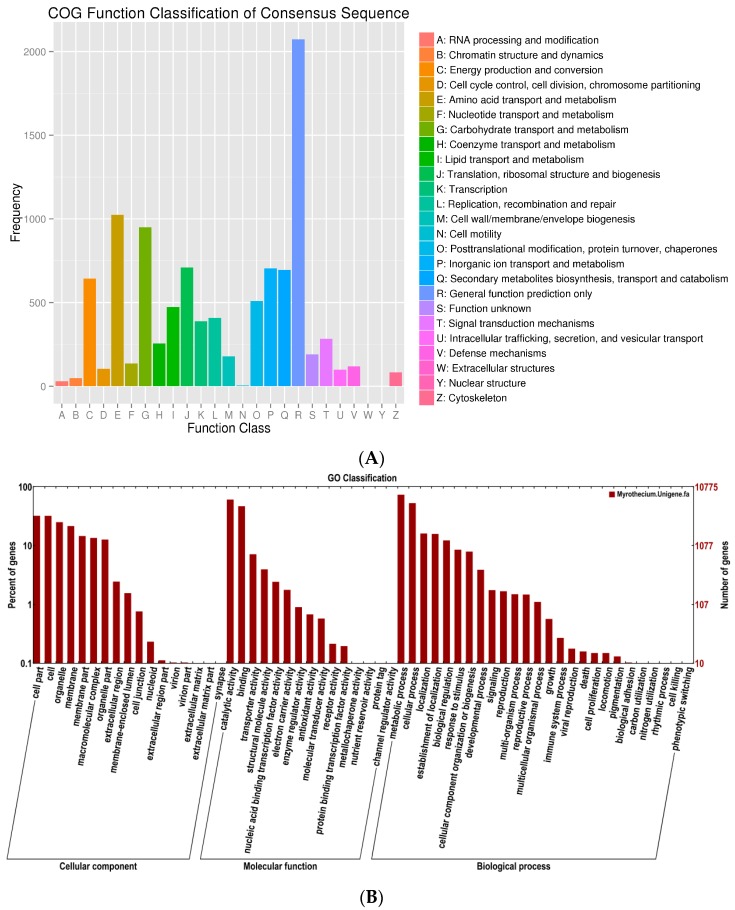
COG function and GO function classification of the *M. roridum* transcriptome. (**A**) COG functional classification of the *M. roridum* transcriptome; (**B**) GO functional classification of the *M. roridum* transcriptome.

A total of 10,113 unigenes were annotated and grouped into 25 functional classifications (Figure 2A). The most frequently identified classes were “general function” (2073, 20.5%), followed by transcription (1024, 10.1%), carbohydrate transport and metabolism (950, 9.4%), translation, ribosomal structure and biogenesis (709, 7.0%), inorganic ion transport and metabolism (704, 6.9%), secondary metabolites biosynthesis, transport, and catabolism (696, 6.8%), and post-translational modification, protein turnover, and chaperones (509, 5.0%). The nuclear structure (1, 0.001%) and extracellular structure (0, 0%) categories showed the fewest corresponding genes. Another 10,775 unigenes were annotated by GO database and classified into the biological process, cellular component, and molecular function categories (Figure 2B). The high percentage of unigenes involved in the function of catalytic activity indicated the variety of the secondary metabolites produced by *M. roridum*. To investigate the biological function of unigenes further, a total of 4569 unigenes were assigned to the metabolic pathways described in the KEGG database, including metabolic pathways, biosynthesis of secondary metabolites, ubiquitin-mediated proteolyis, mitogen-activated protein kinase (MAPK) signaling pathway, and regulation of autophagy RNA transport.

### 2.3. Candidate Genes Involved in Trichothecene Biosynthesis and Expression Analysis of Eight Candidate Genes by qPCR

**Figure 3:**
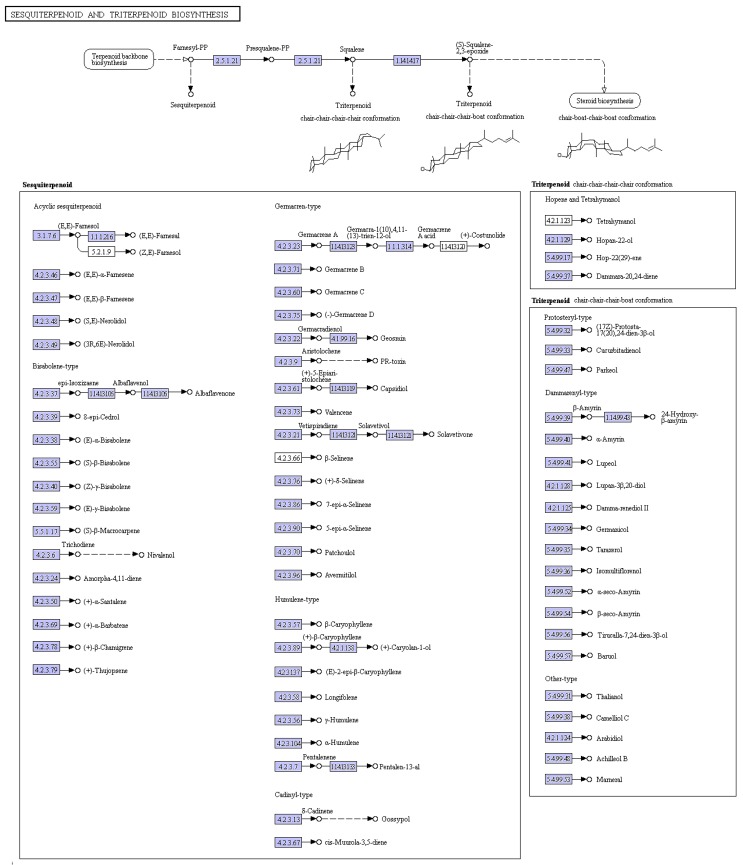
Sesquiterpene and terpenoid backbone biosynthesis pathway in *M. roridum*.

**Figure 4:**
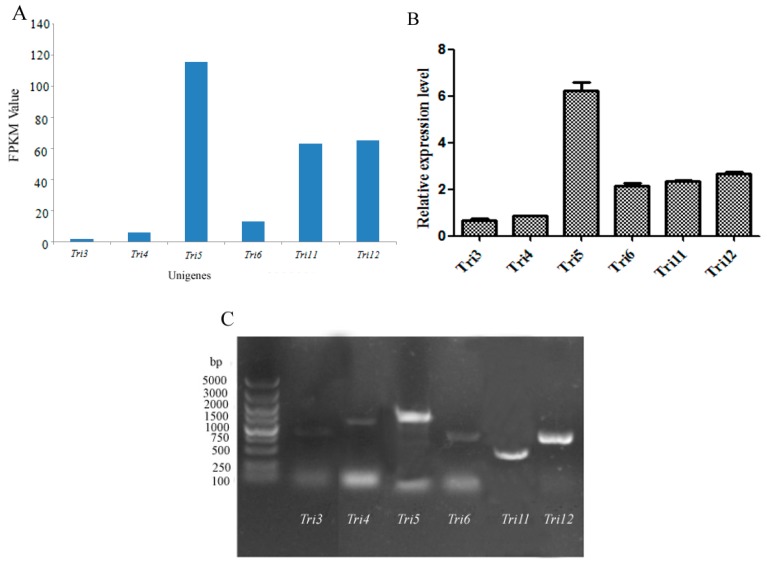
Identification of unigenes involved in trichothecene biosynthesis in *M. roridum*: (**A**) prediction of expression levels of unigenes involved in trichothecene biosynthesis according to the FPKM value; (**B**) validation of expression levels of unigenes involved in trichothecene biosynthesis by qRT-PCR; (**C**) agarose electrophoresis of qRT-PCR products of unigenes involved in trichothecene biosynthesis.

Trichothecene was postulated to play a very important role in the pathogenicity of *M. roridum* [4]. Thus the identification of genes involved in trichothecene biosynthesis establishes a foundation for the elucidation of the pathogenic mechanism of *M. roridum.* Trichothecenes are sesquiterpene compounds, and the sesquiterpene and terpenoid backbone biosynthesis pathways in *M. roridum* are shown in Figure 3. Genes involved in trichothecene mycotoxin biosynthesis are summarized in Table S1. The expression levels of unigenes, including *Tir3* (trichothecene 3-*O*-acetyltransferase), *Tri4* (trichodiene oxygenase), *Tri5* (trichodiene synthase), *Tri6* (trichothecene biosynthesis transcription factor), *Tri12* (trichothecene efflux pump), and *Tri11* (trichothecene 15-*O*-acetyltransferase) were predicted according to the FPKM (Fragments Per Kilobase of exon model per Million mapped reads) value and validated by qRT-PCR (Quantitative real-time polymerase chain reaction) using primers (Table S2) according to the ORFs of the aforementioned genes (Figure 4A,B). *Tri5* gene showed the highest expression level, followed by the genes *Tri12*, *Tri6*, and *Tri11*, suggesting the important roles of these four genes in trichothecene biosynthesis, while *GAPDH* was used as a reference gene. The expression levels identified by qRT-PCR were in accordance with the prediction. The qPCR products of *Tri3*, *Tri4*, *Tri5*, *Tri6*, *Tri11*, and *Tri12* genes were detected by agarose gel (Figure 4C). The sequencing results indicated that the fragments were desired genes involved in trichothecene biosynthesis.

### 2.4. Identification of Tri4, Tri5, and Tri11 Genes

**Figure 5:**
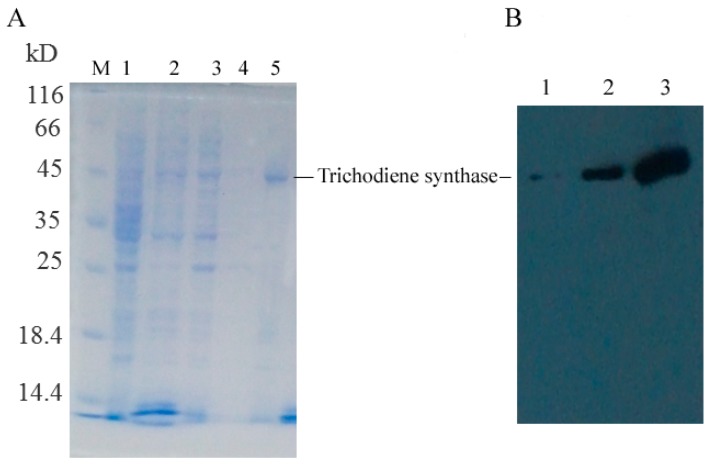
Identification of *Tri5* gene: (**A**) expression and purification of trichodiene synthase encoded by *Tri5* gene from *M. roridum*: M. Protein Marker; 1. Un-induced sample; 2. Total proteins of induced samples; 3. Supernatant of induced sample; 4. 55 mM imidazole eluate; and 5. 100 mM imidazole eluate; and (**B**) Western blot analysis of trichodiene synthase encoded by *Tri5* gene from *M. roridum*: 1. Total proteins of un-induced sample; 2. Supernatant of induced sample; and 3. 55 mM imidazole eluate.

**Figure 6:**
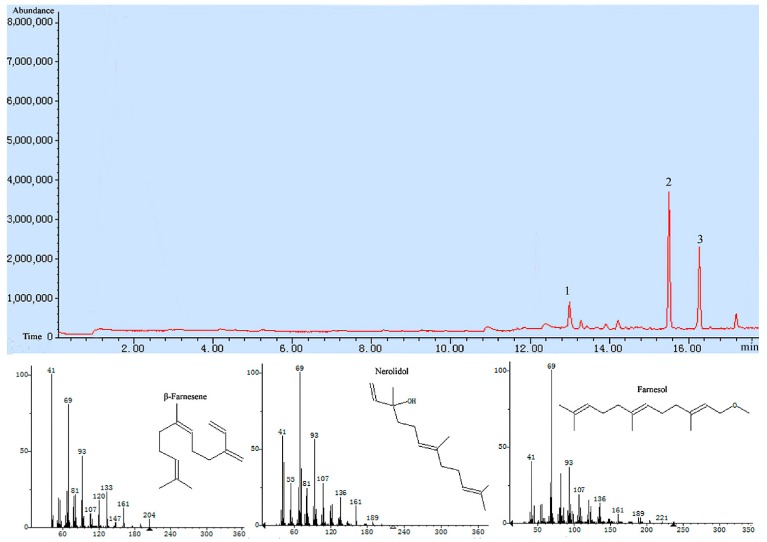
Products catalzyed by trichodiene synthase from *M. roridum* were detected by GC-MS: peaks 1, 2, and 3 corresponded to β-farnesene, nerolidol and farnesol, respectively.

Specific primers were designed to amplify the *Tri4*, *Tri5*, and *Tri11* genes with restriction enzyme sites (*NdeI* and *XhoI*), then the amplified fragment was inserted into vector pET28a and expressed in *Escherichia coli* BL21 (DE3) with a molecular weight of 52.0, 45.0, and 50.0 kDa, respectively, which was in accordance with the theoretical value. The trichodiene synthase was purified by Ni affinity chromatography with a purity of 91.9% (Figure 5A). The Western blot analysis result using anti-His monoclonal antibody confirmed the successful expression and purification of the *Tri5* gene (Figure 5B). Trichodiene synthase from *M. roridum* encoded by *Tri5* gene were added to the substrate FPP, and the catalyzation products were detected by GC-MS (Gas Chromatograph-Mass Spectrometer), and the sesquiterpenoids including β-farnesene, nerolidol and farnesol were detected, nerolidol was detected as a main product, which accounted for 68.2% of all the catalyation products (Figure 6). The result further demonstrated that the trichodiene synthase encoded by *Tri5* was a sesquiterpene synthase.

**Figure 7:**
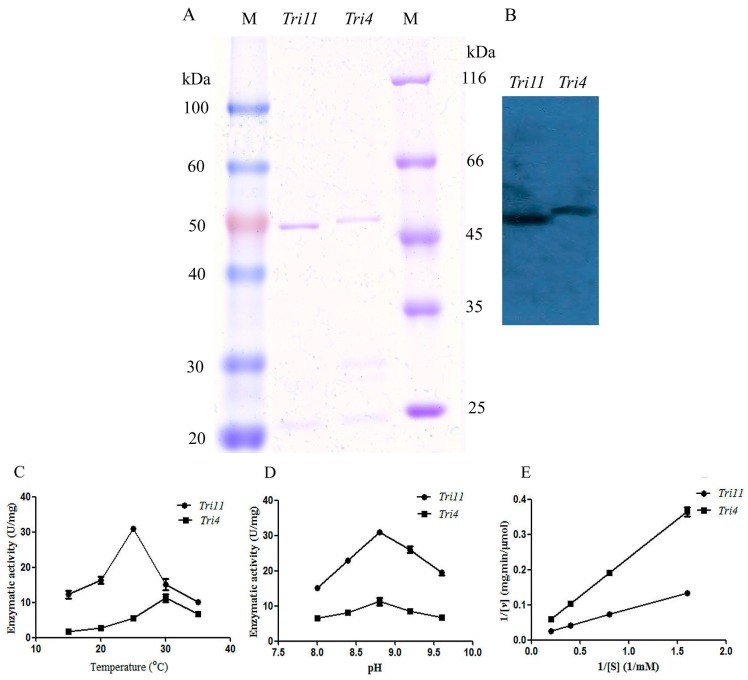
Identification of *Tri4* and *Tri11* genes: (**A**) the expression and purification of enzymes encoded by *Tri4* (TRO-MR) and *Tri11* (ITH-MR), M indicated protein marker; (**B**) the Western blot analysis of TRO-MR and ITH-MR using anti-His monoclonal antibody; (**C**) the optimal reaction temperatures of TRO-MR and ITH-MR; (**D**) the optimal reaction pH values of TRO-MR and ITH-MR; and (**E**) the enzymatic kinetics of TRO-MR and ITH-MR, the *K*_m_ and *V*_max_ values were calculated.

The trichodiene oxygenase encoded by *Tri4* from *M. roridum* (TRO-MR) and isotrichodermin C-15 hydroxylase encoded by *Tri11* from *M. roridum* (ITH-MR) were purified by Ni affinity chromatography with purities of 92.5% and 95.7%, respectively, which was also demonstrated by Western blot analysis (Figure 7A,B). The substrate thioanisole was used to investigate the enzymatic properties of enzymes encoded by *Tri4* and *Tri11.* Trichodiene oxygenase showed the highest enzymatic activity of 11.26 ± 1.53 U/mg towards thioanisole at 30 °C, whereas isotrichodermin C-15 hydroxylase showed the highest enzymatic activity of 31.0 ± 0.6 U/mg towards thioanisole at 25 °C (Figure 7C). The optimal reaction pH values of trichodiene oxygenase and isotrichodermin C-15 hydroxylase from *M. roridum* with thioanisole were 8.8 (Figure 7D). The enzymatic kinetics of TRO-MR and ITH-MR towards thioanisole was investigated by using different substrates (Figure 7E). ITH-MR and TRO-MR showed *V*_max_ and *K*_m_ values of 94.34 μmol/mg∙min, 7.19 mM, and 71.43 µmol/mg∙min, 15.53 mM, respectively. The results indicated that ITH-MR showed higher substrate-binding affinity than TRO-MR.

### 2.5. Development and Characterization of cDNA-Derived SSR Markers

Different unigenes in *M. roridum* were identified by MISA analysis, and 2200 unigenes containing SSR (Simple Sequence Repeat) were identified, among which, 402 sequences contained more than one SSR. Moreover, 202 SSRs were present in the compound formation. The distribution and frequency of the mono-, di-, tri-, tetra-, and hexa-nucleotide repeats were analyzed. The most abundant repeat motifs were mononucleotides (1115, 50.68%), followed by trinucleotides (592, 26.91%), dinucleotides (216, 9.82%), and tetra-nucleotides (48, 2.18%). The most frequent was A/T (904, 41.09%), followed by C/G (340, 15.45%), AGC/CTG (143, 6.5%), AAAC/GTTT (129, 5.86%), AG/CT (128, 5.82%), AAG/CTT (125, 5.68%), AGG/CTT (91, 4.14%), and ACG/CGT (72, 3.27%).

### 2.6. Phylogenetic Analysis of Genes Involved in Trichothecene Mycotoxin Biosynthesis

*Tri5* gene, encoding trichodiene synthase in *M. roridum*, played a very important role in trichothecene biosynthesis. Therefore, phylogenetic analysis was conducted using an alignment of all known predicted protein sequences in the NCBI database (Figure S1A), and the result demonstrated that the highest similarity of trichodiene synthase from *M. roridum* (c10283) with that from *Fusarium sporotrichioides* was only 26%, indicating significant differences between the trichodiene synthase from *M. roridum* and the trichodiene synthases from other fungal species. *Tri4* was annotated as the trichodiene oxygenase from *F. sporotrichioides* in the Nr database. It shared a similarity of 32% with the cytochrome P450 protein from *Eutypa lata*, and nine kinds of trichodiene oxygenases were found in *M. roridum*, exhibiting low similarities (Figure S1B). The alignment results demonstrated the diversity and novelty of trichodiene oxygenases from *M. roridum*.

### 2.7. Construction of Protein–Protein Interaction Network

**Figure 8:**
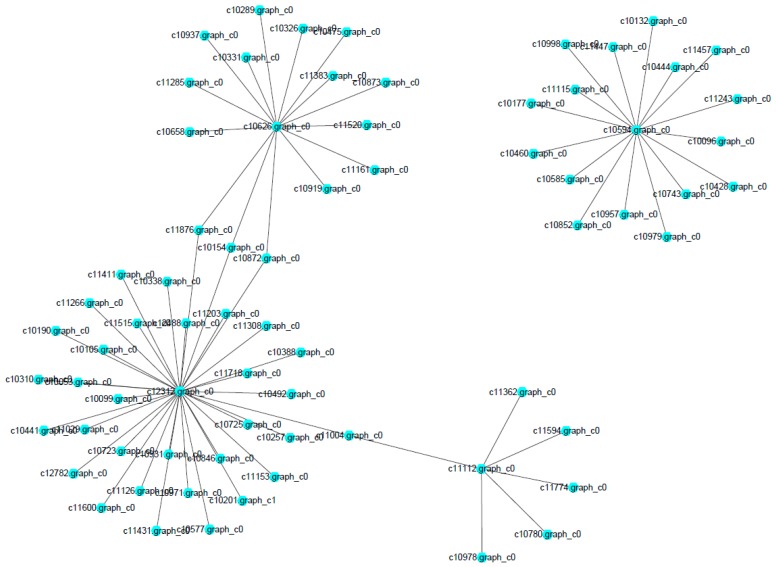
Protein interaction networks in *M. roridium* transcriptome.

The network of protein–protein interaction involved in the MAPK signaling pathway and ribosome biogenesis in *M. roridum* was constructed (Figure 8). A total of 54 and 17 unigenes were involved in the MAPK signaling pathway and ribosome biogenesis, respectively. The two pathways played very important roles in the growth and virulence of *M. roridum.* Unigene c10594 encoding U3 small nucleolar RNA-associated protein 7 is the central unigene for the network of ribosome biogenesis in *M. roridum*. The Unigene 10626 encoding CDC42-GTP-binding protein of the Ras superfamily, unigene c12312 encoding ubiquitin, and unigene c11112 encoding transport protein SEC23 are the central unigenes for the network of MAPK signaling pathway in *M. roridum.*

## 3. Discussion

*M. roridum* is a plant pathogenic fungus that causes great loss to economical crops. The pathogenic mechanism remains unclear. Fungi of *Collecotrichum*, *Nemania*, *Xylaria*, *Phomopsis*, and *Alternaria* isolated from leaves of *Ageratina adenophora* showed strong pathogenicity towards 11 kinds of native plants and four types of economical crops [14]. *Phyllosticta capitalensis*, a kind of plant endophyte, displayed pathogenicity towards various plant families [15]. The transcriptome analysis is an important approach to reveal the molecular pathology of plant pathogenic fungi. *Rhizoctonia solani* is an important plant pathogen, which cause disease in many crops as well as ornamental plants and forest trees. The transcriptome analysis of *R. solani* and the identification of pathogenicity-related genes revealed that genes encoding cellulose, pectin and lignin degrading enzymes and genes related to the MAPK pathway played important roles in the pathogenicity of *R. solani* [13].

In our previous work [16], three trichothecene compounds were obtained from the fermentation liquid of *M. roridum*. In this study, de novo transcriptome analysis of *M. roridum* isolated from *Pogostemon cablin* was performed to investigate the pathogenic mechanism and genes associated with trichothecene mycotoxin biosynthesis. A total of 4569 unigenes were assigned to 104 KEGG pathways, and 202 SSRs were identified. Our *M. roridum* transcriptome results revealed that 47 unigenes, which encoded key enzymes in the trichothecene biosynthesis, including *Tri3*, *Tri4*, *Tri5*, *Tri6*, *Tri11*, and *Tri12*, were postulated according to the biosynthesis pathway of trichothecene mycotoxins in *Fusarium* spp. qPCR analysis was employed to validate the expression levels of these unigenes. PCR and sequencing were performed to confirm the results of the transcriptome analysis. qRT-PCR analysis results revealed that *Tri5* gene showed the highest expressed level, *Tri12*, *Tri6* genes took the second and the third positions, respectively. These results demonstrated the important role of *Tri5* gene in the biosynthesis of trichothecene mycotoxins. The phylogenetic analysis of genes *Tri4* and *Tri5* revealed that significant differences between the trichothecene-biosynthesis-related unigenes from *M. roridum* and those from other fungal species existed. The identification of *Tri5* gene from *M. roridum* confirmed that the function of *Tri5*-encoding protein as a sesquiterpene synthase, meanwhile, trichothecene mytoxins are known as a kind of sesquiterpenoids, thus demonstrating the important role of *Tri5* gene in the biosynthesis of trichothecene mytoxins. The identification of *Tri4* and *Tri11* genes from *M. roridum* demonstrated the function of *Tri4* and *Tri11* as monooxygenase, which played an important role in the biosynthesis of trichothecene mytoxins. The transcriptome analysis of *M. roridum* and identification of the genes related to trichothecene biosynthesis establishes a foundation for the elucidation of molecular plant pathology of the *M. roridum.*

Eleven unigenes, which were found in the transcriptome of *M. roridum*, were annotated as *Tri4* gene encoding trichodiene oxygenase. Different *Tri4* unigenes showed significant differences between each other, indicating that different kinds of trichodiene oxygenases in *M. roridum* contributed to the biosynthesis of different secondary metabolites. Epiroridin acid, epiroridin E, and mytoxin B were isolated from the fermentation liquid of *M. roridum* [16] (Figure S2), and trichodiene oxygenase was assumed to play an important role in the transformation of epiroridin E into mytoxin B. Only one unigene encoding trichodiene synthase was annotated in the *M. roridum* transcriptome, and this trichodiene synthase showed the highest similarity of 26% with that from the *F. sporotrichioides*, suggesting that unigene c3810 encodes a novel trichodiene synthase. The three trichothecene compounds, epiroridin E, epiroridin acid, and mytoxin B, isolated from *M. roridum* showed different structures with known trichothecene mycotoxins, such as T-2 and DON toxin, indicating the possible special biosynthesis mechanism of trichothecens in *M. roridum.* Moreover, the amino acid sequence of regulatory protein encoded by the *Tri6* gene from *M. roridum* showed the highest similarity of 65% with that from *F. sporotrichioides*, implying the possibly different regulatory mechanism of trichothecene biosynthesis in *M. roridum.*

The *stel2* gene involved in the MAPK signal pathway in opportunistic pathogenic fungus *Cryptococcus neoformans* was reportedly capable of regulating the expression of virulence-related genes, including capsule and melanin-related genes [17,18], regulating the virulence of *C. neoformans*. MAPK signal pathway was also reported to be involved in the process of phenotype switching and mycelium formation, which played an important role in the invasive infection of *Candida albicans* [19]. Thus, the MAPK signal pathway, especially the transport protein SEC23, is assumed to have played an important role in the pathogenicity of *M. roridum.*

## 4. Materials and Methods

### 4.1. cDNA Library Construction and RNA Sequencing

The strain *M. roridum* A553 (Accession No. KJ813720) was isolated from the medicinal plant *Pogostemon cablin*, a kind of Guangdong medicinal plant collected from Xinyi City, Guangdong province. *M. roridum* was inoculated on a PDA medium and incubated at 30 °C for 3, 5, 7, and 9 days. The total RNAs of *M. roridum* at different growth stages were determined using the RNA extracting kit (Umagen, Guangzhou, China). The quantity, purity, and integrity of RNA were checked on a 1.5% (*w*/*v*) agarose gel and the Nanodrop-2000 spectrophotometer (GE, Fairfield, CT, USA). HPLC (High Performance Liquid Chromatography) Agilent 2100 (Agilent, Santa Clara, CA, USA) was used to detect the RIN value of the total RNA. High-quality samples (RNA ≥ 6.0) were selected for high-throughput sequencing. Then, the different RNAs were mixed with the same amount. Total RNAs in an amount of 5.0 µg were resuspended in RNase free-water and stored at −80 °C until use. The extracted RNA samples were used for the complementary DNA (cDNA) synthesis. Poly(A) mRNA was isolated using oligo-dT beads (Qiagen, Germantown, MD, USA). All mRNAs were broken into short fragments (200 nt) by adding a fragmentation buffer. First-strand cDNA was generated using random hexamer-primed reverse transcription, followed by the synthesis of the second-strand cDNA using RNase H and DNA polymerase I. The cDNA fragments were purified using a QIAquick PCR extraction kit (Qiagen, Germantown, MD, USA). These purified fragments were then washed with Elution buffer for end reparation poly(A) addition and ligated to sequencing adapters. Following the agarose gel electrophoresis and extraction of cDNA from gels, the cDNA fragments (200 ± 25 bp) were purified and enriched by PCR to construct the final cDNA library. The cDNA library was sequenced on the Illumina sequencing platform (Illumina HiSeq™ 2000, Illumina, San Diego, CA, USA) using the single-end paired-end technology in a single run. The original image processes of sequencing, base-calling, and quality value calculation were performed by the Illumina GA Pipeline (version 1.6, Illumina, San Diego, CA, USA), in which 90-bp paired-end reads were obtained [20,21].

### 4.2. Data Processing, Assembly, and Annotation

Transcriptome sequencing was conducted using the Illumina HiSeq™ 2000 sequencing platform. To assemble the entire transcriptomes of the different samples better, a paired-end (PE)100 sequencing strategy was used. All sequences were examined to ensure their accuracy. A Perl program was written to select clean reads by removing low-quality sequences (more than 50% bases with quality lower than 20 in one sequence and Q30 less than 80% were identified), reads with more than 5% N bases (bases unknown), and reads containing adaptor sequences. Subsequently, the clean reads were assembled using the Trinity software (version 1.4, Campton, NH, USA) to construct unique consensus sequences. Adaptor and low-quality sequences were trimmed. Short sequences (<50 bp) were removed using a custom Perl program (version 5.14, USA). The resulting high-quality sequences were deposited in the National Center for Biotechnology Information (NCBI) database and de novo assembled into contigs and transcripts. To reduce data redundancy, transcripts with a minimum length of 200 bp were assembled and clustered using the CLC NGS Cell software (version 1.3, Illumina, San Diego, CA, USA) under default parameters. The longest sequences in each cluster were reserved and designated as unigenes. Searches were performed using local BLASTX programs (NCBI, NIH, Bethesda, MD, USA) against sequences in the NCBI non-redundant (nr) protein database and the SWISS-PROT database (the *e* value cut-off was 1 × 10^−5^) [22]. Unigenes were tentatively identified according to top hits against known sequences. The resulting unigenes were used as references for the determination of GO and COG terms and were analyzed further using the KEGG database.

### 4.3. KEGG Pathway Analysis and Predicted CDS

Pathway assignments were made according to KEGG mapping. Enzyme commission numbers were assigned to unique sequences that had the best BLASTX scores with cutoff *e* values of 1.00 × 10^−5^, as determined from our KEGG database search. The KEGG database is capable of analyzing gene products during the metabolism process and related gene function in the cellular processes. The sequences were mapped to the KEGG biochemical pathways according to the Enzyme Commission (EC) distribution within the pathway database.

Then, we used blast results information to extract CDS (Coding Domain Sequences) from Unigene sequences and translated them into peptide sequences. Moreover, blast results information was also used to train ESTScan [23]. The CDS of unigenes that had no hit in BLAST were predicted by ESTScan and then translated into peptide sequences.

### 4.4. Quantitative Real-Time Polymerase Chain Reaction Analysis

To verify the quality of the sequences assembled in this study, unigenes related to the biosynthesis of trichothecene were validated by quantitative real-time polymerase chain reaction (qRT-PCR). qRT-PCR was performed using the Mastercycler ep realplex System (Eppendorf, Westbury, NY, USA) with 2SYBR Green mix (Fermentas, Harrington, DE, USA) according to the manufacturer’s instructions. First-strand cDNA was synthesized from 1 μg of total RNA with reverse transcriptase (Takara, Tokyo, Japan) and oligo (dT) 15 primer, and the resulting products were used as templates for qRT-PCR. The specific primers used for qRT-PCR are listed in Table S2. The qRT-PCR thermal cycling condition for all reactions was 95 °C for 1 min 50 s, followed by 40 cycles at 95 °C for 10 s and 55 °C for 33 s. All reactions were conducted in triplicates, and the results were expressed as relative expression levels to the *GAPDH* gene. The *C*_t_ values obtained were used as the original data to calculate the relative expression level of different genes to histone gene by the 2^−^^ΔΔ*C*t^ method [24,25]. Each sample was analyzed in triplicate.

### 4.5. Identification of Tri4, Tri5, and Tri11 Genes

The *Tri4*, *Tri5*, and *Tri11* genes were amplified with restriction enzymes *NdeI* and *XhoI* using primers listed in Table S3, then inserted into the expression vector pET28a and transformed with *E. coli*, and the recombinant vector pET28a-*Tri4*, pET28a-*Tri5*, and pET28a-*Tri11* were expressed in *E. coli* BL21 (DE3) after being induced using 1 mM of IPTG for 3.0 h. A supernatant of 200 mL fermentation liquid containing pET28a-*Tri5* after sonication was loaded onto the Ni affinity chromatography. The target protein was eluted with 20 mM Tris–HCl (pH 8.0) containing different concentrations of imidazole. Different protein samples were identified by sodium dodecyl sulfate-polyacrylamide gel electrophoresis and transferred to the NC membrane. After being blocked by 5% non-fat milk, the membrane was incubated with mouse anti-His monoclonal antibody (Earthox, Millbrae, CA, USA) and goat anti mouse IgG antibody (Promega, Madison, WI, USA), and target bands were finally visualized using an ECL kit (Fermentas, Harrington, DE, USA) following the manufacturer’s instructions.

### 4.6. Enzymatic Activity Assay of Enzymes Encoded by Tri4, Tri5, and Tri11

Briefly, farnesyl pyrophosphate (FPP) substrate with a concentration of 46 µM and 50 µL *Tri5* encoding-protein with a concentration of 0.6 mg/mL were added to a buffer containing 25 mM Tris-HCl (pH 7.0), 100 mM MgSO4, 10% glycerol, and 5 mM DTT. A 200 µL aliquot of the mixture was immediately sealed and incubated at 37 °C for 1 h. A solid phase extraction column (Anpel, Shanghai, China) was used to absorb sesquiterpenes produced by catalysis of *Tri5* encoding-protein at 65 °C for 0.5 h. The absorbed products were loaded onto a HP 6890/5975C GC-MS apparatus (Agilent, Santa Clara, CA, USA). The GC-MS running procedure is as following: the injection port temperature is 250 °C, the initial temperature is 80 °C, increased to 180 °C with a gradient of 5 °C/min, then increased to 250 °C with a gradient of 20 °C/min. The flow rate of helium is 1.0 mL/min. The structure and abundance of the produced sesquiterpenes were analyzed.

NADPH with content of 0.40 µmol and 0.04 mg enzymes were added into substrate thioanisole with a concentration of 1.0 µmol, 50 mM Tris-HCl (pH 8.8) was added to total volume of 200 µL. The mixture was incubated at 25 °C, the increase or the decrease in absorbance at 340 nm were monitored. 1 U was defined as 1 µmol NADPH was consumed in one minute. The optimal reaction temperature and reaction pH for trichodiene oxygenase encoded by *Tri4* and isotrichodermin C-15 hydroxylase encoded by *Tri11* were investigated under the reaction temperatures of 15 °C, 20 °C, 25 °C, 30 °C, 35 °C and reaction pH values of 8.0, 8.4, 8.8, 9.2, and 9.6. Substrate thioanisole with different concentrations of 5.0 mM, 2.5 mM, 1.25 mM, and 0.625 mM were used to investigate the enzymatic kinetics of enzymes encoded by *Tri4* and *Tri11* genes.

### 4.7. Phylogenetic Analysis of Genes Related to Trichothecene Mycotoxin Biosynthesis

A phylogenetic tree was constructed with MEGA version 5.0 (Arizona State University, Tempe, AZ, USA) using the neighbor-joining method [26]. NCBI and the transcriptome of *M. roridum* were searched for different trichodiene synthases and trichodiene oxygenases. The functional interaction networks of proteins were integrated using the STRING (Search Tool for the Retrieval of Interacting Genes/Proteins) database with the confidence parameter set at 0.15 threshold. The expression clusters of the *CsWRKY* genes from each cultivar were analyzed using Cluster (http://bonsai.hgc.jp/~mdehoon/software/cluster/software.htm). A diagram was drawn using Tree View (http://jtreeview.sourceforge.net/).

### 4.8. Identification of SSRs

The MIcroSAtellite identification tool (MISA, http://pgrc.inpk-gatersleben.de/misa/) was used to identify SSRs [27]. The parameters were adjusted to identify perfect dinucleotide, trinucleotide, tetranucleotide, pentanucleotide, and hexanucleotide motifs with a minimum of 6, 5, 5, 4, and 4 repetitions, respectively. Primer pairs were designed using the Primer 3 software and selected according to the following criteria: (1) primers with SSRs were eliminated; (2) primers aligned with unigene sequences were allowed three mismatches at the 5′ site and one mismatch at the 3′ site; (3) primers that aligned to more than one unigene were eliminated; and (4) SSRs were identified using the ssr_finder (http://www.fresnostate.edu/ssrfinder/). We kept the products whose results from ssr_finder and MISA were the same.

## 5. Conclusions

In conclusion, the transcriptome of *M. roridum* was first sequenced by an Illumina Hiseq 2000 system. The expression levels of genes related to the trichothecene biosynthesis were analyzed, and several genes related to the biosynthesis of trichothecene were cloned and expressed. The results demonstrated that *Tri5* gene played an important role in the biosynthesis of trichothecene mycotoxins in *M. roridum.* The enzymatic properties investigation results demonstrated that *Tri4* and *Tri11* genes’ function as monooxygenase in the biosynthesis of trichothecene mycotoxins. The protein–protein network of the *M. roridum* transcriptome indicated that the MAPK signal pathway also played a role in the pathogenicity of *M. roridum*. The above results establish a foundation for the elucidation of the trichothecene biosynthesis and molecular mechanism of *M. roridum* pathogenicity, thus providing a molecular basis for controlling *M. roridum* infection through genetic engineering approaches.

## Figures and Tables

**Table 1:** Raw data of *M. roridum* transcriptome.

Sample ID	Readsum	Basesum	GC (%)	N (%)	Q20 (%)	Q30 (%)
T01	44992952	8997597886	57.57	0.01	92.49	85.07

**Table 2:** All unigenes of *M. roridum* transcriptome annotated in different databases.

Anno_Database	Annotated_Number	300 ≤ Length < 1000	Length ≥ 1000
COG_Annotation	7046	2026	3842
GO_Annotation	10,775	3400	4730
KEGG_Annotation	4569	1361	2134
Swissprot_Annotation	11,376	3158	6458
nr_Annotation	18,160	6034	8127
All_Annotated	18,199	6046	8128

## Supplementary Materials

Supplementary materials can be found at www.mdpi.com/1422-0067/18/3/497/s1.

## Abbreviations

CDS	Coding Domain Sequences
COG	Cluster of Orthologous Groups of proteins
FPKM	Fragments Per Kilobase of exon model per Million mapped reads
FPP	Farnesyl pyrophosphate
GC-MS	Gas Chromatograph-Mass Spectrometer
GO	Gene Ontology Consortium
HPLC	High Performance Liquid Chromatography
ITH-MR	Isotrichodermin C-15 hydroxylase from *M. roridum*
KEGG	Kyoto Encyclopedia of Genes and Genomes
MAPK	Mitogen-activated protein kinase
qRT-PCR	Quantitative real-time polymerase chain reaction
SSR	Simple Sequence Repeat
STRING	Search Tool for the Retrieval of Interacting Genes/Proteins
TRO-MR	Trichodiene oxygenase from *M. roridum*
