# Supplementary material for: De Novo Transcriptome Analysis of Plant Pathogenic Fungus Myrothecium roridum and Identification of Genes Associated with Trichothecene Mycotoxin Biosynthesis
Source: Int J Mol Sci. 2017 Feb 25;18(3):497. doi: 10.3390/ijms18030497 (PMC5372513; doi:10.3390/ijms18030497)
Supplement: Supplementary file 1 [file ijms-18-00497-s001.pdf]

Supplementary Materials: De Novo Transcriptome Analysis of Plant Pathogenic Fungus *Myrothecium roridum* and Identification of Genes Associated with Trichothecene Mycotoxin Biosynthesis

Wei Ye, Taomei Liu, Muzi Zhu, Weimin Zhang, Haohua Li, Zilei Huang and Saini Li

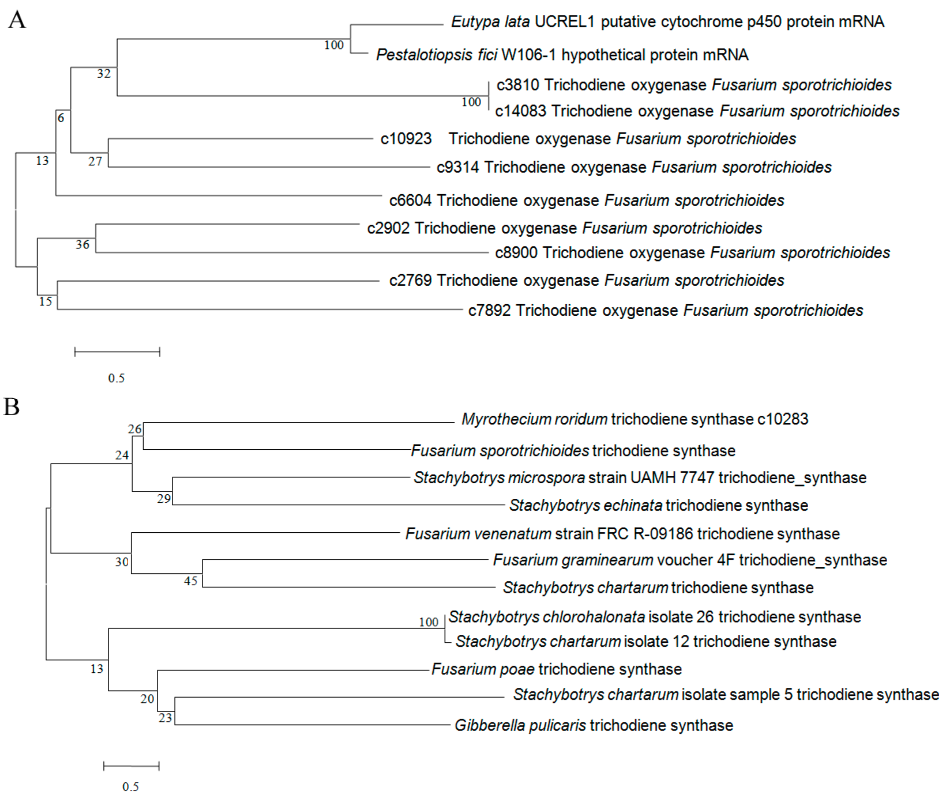

Figure S1. The phylogenetic tree analysis of *Tri4* and *Tri5* gene in *Myrothecium roridium.*

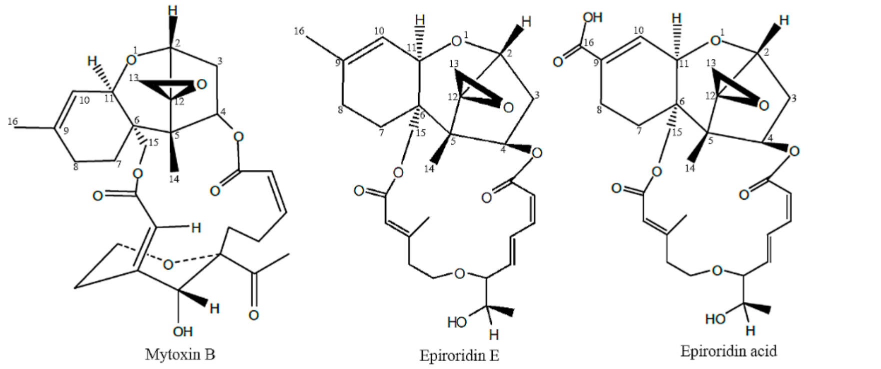

Figure S2. The structure of trichotecene mycotoxins isolated from *Myrothecium roridium.*

Table S1. Unigenes involved in the biosynthesis of trichothecenes in *M. roridum.*

#Gene ID	Swissprot_annotation
c9314.graph_c0	Trichodiene oxygenase GN=TRI4 OS=*Fusarium sporotrichioides* PE=3 SV=1
c21357.graph_c0	Isotrichodermin C-15 hydroxylase GN=TRI11 OS=*Fusarium sporotrichioides* PE=3 SV=1
c15279.graph_c0	Isotrichodermin C-15 hydroxylase GN=TRI11 OS=*Fusarium sporotrichioides* PE=3 SV=1
c6640.graph_c0	Trichodiene oxygenase GN=TRI4 OS=*Fusarium sporotrichioides* PE=3 SV=1
c12743.graph_c0	Isotrichodermin C-15 hydroxylase GN=TRI11 OS=*Fusarium sporotrichioides* PE=3 SV=1
c5491.graph_c0	Isotrichodermin C-15 hydroxylase GN=TRI11 OS=*Fusarium sporotrichioides* PE=3 SV=1
c12125.graph_c0	Isotrichodermin C-15 hydroxylase GN=TRI11 OS=*Fusarium sporotrichioides* PE=3 SV=1
c22090.graph_c0	Isotrichodermin C-15 hydroxylase GN=TRI11 OS=*Fusarium sporotrichioides* PE=3 SV=1
c25252.graph_c0	Isotrichodermin C-15 hydroxylase GN=TRI11 OS=*Fusarium sporotrichioides* PE=3 SV=1
c5491.graph_c1	Isotrichodermin C-15 hydroxylase GN=TRI11 OS=*Fusarium sporotrichioides* PE=3 SV=1
c21206.graph_c0	Isotrichodermin C-15 hydroxylase GN=TRI11 OS=*Fusarium sporotrichioides* PE=3 SV=1
c13046.graph_c0	Isotrichodermin C-15 hydroxylase GN=TRI11 OS=*Fusarium sporotrichioides* PE=3 SV=1
c12970.graph_c0	Isotrichodermin C-15 hydroxylase GN=TRI11 OS=*Fusarium sporotrichioides* PE=3 SV=1
c9277.graph_c0	Trichodiene oxygenase GN=TRI4 OS=*Fusarium sporotrichioides* PE=3 SV=1

Table S2. Primers used for the qRT-PCR of genes related to the trichothecene biosynthesis in *M. roridum.*

Genes	Sequences (5′–3′)
Tri3 F	TTCCATCCATGGCAGACTTG
Tri3 R	ATCCATCTCGTAGGAGTCC
Tri4 F	GCGGCTGCCACATATTTGCCCG
Tri4 R	TGGTTGTGATAAAAGGAAGTAGG
Tri5 F	TTTCTGCCCAGAAATATCTATC
Tri5 R	ACGGTGAGCTTCTCCAGAGCCTGG
Tri6 F	GGCTTCTTCCACGACTATGAAATG
Tri6 R	CCTCCGTTCCGATCTTGCCA
Tri11 F	ATGCCCGTTTTGAGAACC
Tri11 R	CAGGCTCCTTCTCCCTTG
Tri12 F	ATCGCTTCGGTCGCCGGC
Tri12 R	TTCCGGAAGCCCAGCCTC

Table S3. Primers used for the amplification of genes related to the trichothecene biosynthesis in *M. roridum.*

Genes	Sequences (5′–3′)
Tri4 F	GGAATTCCATATGGCTGTTCCAGAGGATGTG
Tri4 R	CCGCTCGAGCTTCTCCTCTGCGTACACC
Tri11 F	GGAATTCCATATGCGCTATGGCCCAAATCG
Tri11 R	CCGCTCGAGTCCACGAGACGTGAAGTTG
Tri5 F	GGAATTCCATATGTTTCTGCCCAGAAATATCTATC
Tri5 R	CCGCTCGAGACGGTGAGCTTCTCCAGAGCCTGG
